# Pattern of Virtual Consultations in the Kingdom of Saudi Arabia: An Epidemiological Nationwide Study

**DOI:** 10.1007/s44197-024-00219-3

**Published:** 2024-04-04

**Authors:** Reem S. AlOmar, Muaddi AlHarbi, Nijr S. Alotaibi, Nouf A. AlShamlan, Malak A Al-Shammari, Arwa A. AlThumairi, Mona AlSubaie, Mohammed A. Alshahrani, Mohammad K. AlAbdulaali

**Affiliations:** 1 Department of Family and Community Medicine, College of Medicine, Imam Abdulrahman Bin Faisal University, P.O. Box 1982, Dammam, 32211 Kingdom of Saudi Arabia; 2 The Studies and Consulting Office at the Assistant Minister of Health, Ministry of Health, Riyadh, Kingdom of Saudi Arabia; 3 National Program for Community Development – Tanmiah, Riyadh, Kingdom of Saudi Arabia; 4 Department of Health Information Management and Technology, College of Public Health, Imam Abdulrahman Bin Faisal University, Dammam, Kingdom of Saudi Arabia; 5 Virtual Hospital, Ministry of Health, Riyadh, Kingdom of Saudi Arabia; 6 Ministry of Health, Riyadh, Kingdom of Saudi Arabia; 7 Human Resources and Training Management, Ministry of Health, Riyadh, Saudi Arabia

**Keywords:** Epidemiology, Public health, Public health policy, Health care systems, Virtual consultations

## Abstract

**Background:**

In the Kingdom of Saudi Arabia (KSA), little is known about the adoption of virtual consultations (VCs), with most studies being survey-based leading to varying results. This study aims to utilise secondary collected data on the use of both kinds of VCs currently available, and to epidemiologically describe the adoption of these consultations.

**Methods:**

This retrospective study analysed data provided by the Ministry of Health between January 1st 2021 and June 30th 2022. For both the home-based and the hospital-based consultations, variables included the age and sex of patients, date of consultation, duration in minutes, closure status for the appointment and the governorate in which the patient is residing. A heat map was drawn to present patterns of utilisation across the country.

**Results:**

The total number of VCs for both types were 1,008,228. For both types, females were higher adopters (54.73%). Of the total number of consultations, 751,156 were hospital-based. Of these consultations, family medicine consultations were the most common (20.42%), followed by internal medicine. Maternity follow-up clinics were higher in home-based clinics. The proportion of patient no-shows was high overall (48.30%). Utilisation was high in urban governorates, and low in rural ones.

**Conclusion:**

Findings have several implications on health policy. It provides further evidence of the importance of family medicine, where it was the most common speciality even in hospital-based settings. The high variability in the adoption of consultations across rural and urban areas as well as the extremely high number of patient-no-shows warrants further investigation.

## Background

In the Kingdom of Saudi Arabia (KSA), the Ministry of Health (MoH) is responsible for strategic health planning, formulating health policies, managing health service delivery programs, and supervising all other health-related activities in both the public and private sectors [1]. In line with the Saudi government’s Vision 2030 and the National Transformation Program, the MoH is expected to spend close to $71 billion to reconfigure its existing healthcare system to improve the quality of primary, preventive and therapeutic healthcare services focusing on digital healthcare innovations [2]. The Saudi healthcare system is currently in a significant transformational phase towards privatisation. Despite the benefits of having strategic plans in place, the complexities associated with a transitioning healthcare system presents significant challenges. Subsequently, there is a need to identify more ways to manage these complexities and to improve productivity.

Healthcare systems remain complex and face similar challenges and the Saudi healthcare system is not immune to them [3]. Therefore, the integration of healthcare delivery via virtual consultations (VCs) (i.e., including synchronous telephone consultations, video, text or image messaging and asynchronous email consultations) is crucial for improving quality; tackling complexity, reducing costs; and delivering safe, timely, efficient, effective, equitable and patient-centred care [4]. This concept emerges from the view that integrated health systems provide superior performance as a result of effective communication and standardised protocols [5].

In the KSA, and as part of the ever-evolving healthcare system, telemedicine tools have been introduced by the MoH [6]. These include direct phone consultations through the Medical Call Centre (937), where primary care physicians are available around the clock to provide consultations according to international guidelines and provide residents with advice for poisoning cases. Also, online consultations are now available through the Sehhaty mobile application. Through this application, patients are able to schedule appointments with the closest primary healthcare centre, initiate virtual consultations, review sick leaves and medical reports, record children’s vaccinations and even provide early weather warning for asthmatics. Additionally, the Anat digital platform allows the health practitioner to schedule appointments for patients if needed and prescribe medications [7–9].

Evidence demonstrates a positive correlation between VCs and various beneficial outcomes such as convenience and timeliness [10]. VCs offer numerous advantages that contribute to improved patient experiences, accessibility, and engagement [11]. However, there is limited existing evidence supporting the implementation of VCs, primarily due to a lack of understanding regarding barriers and potential disadvantages faced by vulnerable and hard-to-reach groups. Consequently, it is important to investigate the pattern of primary care consultations and be informed on sociodemographic differences among users to comprehensively understand and thoroughly evaluate the successful integration of VCs into the current healthcare system. Therefore, the aim of this study is to explore the pattern of primary care consultations in the KSA and identify trends between January 2021 and June 2022. Specifically, it will compare consultations delivered by primary care/family medicine physicians and other specialties with a particular focus on regions.

## Methods

### Study Design and Setting

This retrospective epidemiological study utilised data from the MoH on two types of VCs, namely, home-based consultations, and hospital-based consultations. Home-based consultations are consultations initiated by a patient from their home through a mobile application known as Sehhaty. This application allows patients to schedule an appointment with a primary care/family medicine physician from the nearest primary care centre. The application also allows patients to be referred to a hospital with which the centre is affiliated if the need arises. Whereas in hospital-based consultations, the appointment is initiated by the treating physician from the hospital through the Anat digital platform. Both platforms are under the umbrella of the MoH [6].

### Ethical Considerations

The MoH central institutional review board and the Imam Abdulrahman Bin Faisal University’s institutional review board have both approved the study (IRB log No: 23–39 E and IRB-2022-01-261). Following approval, requested data were sent to authors for analysis. The data did not include any personally identifiable information, and the data was secured and used only for the purposes of this research.

### Study Variables

The data covered an 18-month period between January 1st 2021 and June 30th 2022. The variables included for both types of consultations were age and sex of patients, the month and year of the consultation, and the duration of consultations in minutes. The closure status of the appointment as well as the type of primary care clinic were available for both types of consultations, however some statuses only applied to either the hospital-based or the home-based consultations, e.g. “hospital referral” and “follow-up required” statuses did not apply to home-based consultations and the status of “appointment not planned” did not apply to home-based consultations. Specific to hospital-based consultations, the speciality was used to enable comparisons between primary care/family medicine and other specialities. Additionally, specific to home-based consultations, the governorate from which the patient initiated the appointment was also collected.

### Data Analyses

All data were checked for missing observations, none were found. Data were described as means ± standard deviations for normally distributed continuous variables, and median and interquartile ranges (IQR) for non-normally distributed continuous variables. Frequencies and percentages were computed for categorical variables. Comparisons and cross tabulations were performed, and p-values were computed by means of a series of Chi-squared tests and t-tests after normality of distributions were checked. Where data was not normally distributed, non-parametric tests were performed. The level of significance was set to less than 0.05 The Stata Statistical Software version 16 was used for all analyses [12]. To further study the distribution of home-based consultations across the governorates of the KSA, the proportion of these consultations were computed per governorate, then divided into quintiles and subsequently a heat map of these quintiles was drawn in ArcGIS [GIS software] version 10.0 [13].

## Results

### Sociodemographic Characteristics of Patients With a Virtual Consultation Appointment

The total number of VCs was 1,008,228. The mean age was 38.50 ± 20.46 years. Females made up 54.73% of all consultations. The overall median duration of consultations was 8 min with an IQR of 4 to 13 min. Around 41% of all consultations were completed successfully, whereas 48.30% were closed due to the patient not showing up. Examining the data by the source of the appointment, 74.50% were hospital-based VCs whereas 25.50% consisted of home-based consultations.

Patients utilising hospital-based consultations were older. In both types of consultations, females were higher users (52.41% for home-based and 55.57% for hospital-based consultations). A difference in the median minutes of consultations was observed, where it was higher for home-based consultations (P < 0.001). For home-based consultations, over a quarter of consultations were completed successfully, whereas 59.33% were closed due to the patient not showing up. With regards to hospital-based consultations, around 46% were completed successfully, and 44.53% were closed due to patient no-shows. Home-based consultations identified patients who needed a follow-up (0.62%) and patients needing either a hospital referral or an internal referral (0.49% and 0.11% respectively) (Table 1).

**Table 1:** Overall sociodemographic and appointment characteristics of virtual consultations between January 2021 and June 2022 in the Kingdom of Saudi Arabia

Characteristics	Total consultations N (%) 1,008,228 (100.00)	Sehhaty (Home based) N(%) 257,072 (100.00)	Hospital-based consultations N(%) 751,156 (100.00)
Age (µ, α)	38.50 (20.46)	37.38 (19.92)	38.89 (20.61)
*P-value*		*< 0.001*	
Sex			
Males	456,443 (45.27)	124,890 (47.59)	333,714 (44.43)
Females	551,778 (54.73)	137,548 (52.41)	417,435 (55.57)
* P-value*		*< 0.001*	
Duration of consultation (Median, IQR)	8 (4–13)	9 (5–14)	7 (4–12)
*P-value*		*< 0.001*	
Closure status			
Completed successfully	412,362 (40.90)	66,406 (25.83)	345,956 (46.06)
Follow-up required	1,598 (00.16)	1,598 (00.62)	0
Hospital referral	1,268 (00.13)	1,268 (0.49)	0
Internal referral	281 (00.03)	281 (0.11)	0
Cancelled*	57,267 (05.68)	26,193 (10.19)	31,074 (04.14)
Patient no-show	486,980 (48.30)	152,514 (59.33)	334,466 (44.53)
Physician no-show	24,335 (02.41)	7,806 (03.04)	16,529 (02.20)
Planned	5,195 (00.51)	1,006 (0.39)	4,053 (00.54)
Not planned	17,780 (01.75)	0	17,780 (02.37)
No action specified	1,298 (00.13)	0	1,298 (00.17)
* P-value*		*< 0.001*	

*Cancelled: By either the physician or the patient

#### Services and Clinics Typically Part of a Primary Care Setting by Type of Consultation

Table 2 shows the distribution of VCs from clinics that are typically a part of a primary care setting. These clinics made up 20.42% of all hospital-based VCs, whereas the entirety of the home-based consultations consisted of these clinics. For both types, primary care/family medicine consultations made up the majority of consultations (97.07% and 79.67% respectively). All other consultations were higher in the hospital-based VCs except for maternity follow-up clinics which were higher in home-based consultations (0.67%).

**Table 2:** Distribution of clinics typically part of a primary care centre

Type of primary care clinic appointment	Home-based virtual clinic (Sehhaty) N (%) 257,072 (100.00)	Hospital-based virtual clinic^‡^ N (%) 153,396 (100.00)
General family medicine/primary care	249,551 (97.07)	122,203 (79.67)
Chronic disease clinic	1,423 (00.55)	3,349 (02.18)
Smoking cessation	4,070 (01.58)	7,028 (04.58)
Maternity follow-up	1,714 (0.67)	79 (00.05)
Pre-marital clinic	0	815 (00.53)
Health promotion	0	1,868 (01.22)
Health education	314 (00.12)	18,054 (11.77)

^‡^Percentages for individual clinics do not add up to 100.00%

### Primary Care/Family Medicine vs. Other Specialties in Hospital-Based Consultations

Of the overall 751,156 hospital-based VCs, 153,396 (20.42%) were covered by primary care/family medicine. Females were higher utilisers of both primary care/family medicine and other specialities consultations, although this was much higher for other specialities (50.83% and 56.79% respectively). Patients were slightly older for primary care/family medicine consultations (Mean age = 39.74). The median duration and IQRs of primary care/family medicine consultations were statistically significantly higher than all other specialities (median = 9, IQR = 5–14, and median = 7, IQR = 4–12 respectively). With regards to closure status, 48.84% of primary care/family medicine consultations were completed successfully compared to 45.34% among other specialities. The proportion of cancelled consultations as well as physician no-show was higher among other specialities (4.49% and 2.39% respectively), whereas patient no-show was higher in primary care consultations (Table 3).

**Fig. 1:**
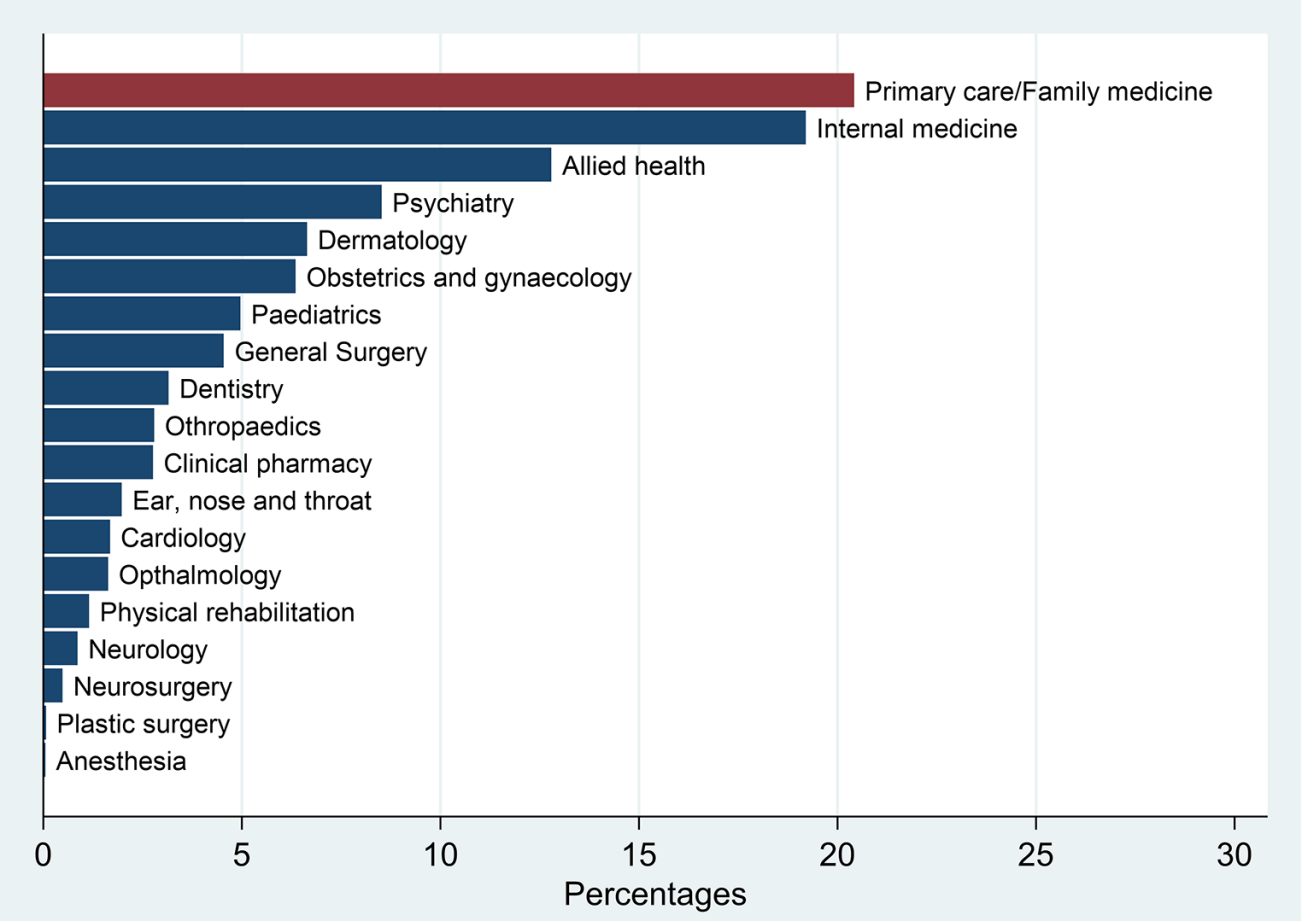
Distribution of all virtual consultations by speciality

The distribution of all hospital-based consultations by speciality shows that primary care/family medicine was the most demanded speciality, followed by internal medicine (Fig. 1).

**Table 3:** Distribution of hospital-based virtual consultations alone

Characteristic	Specialities 751,156 (100.00)		P-value
	Primary care/Family Medicine 153,396 (20.42)	All other specialties 597,760 (79.58)	
Age (µ, α)	39.74 (20.06)	38.68 (20.75)	< 0.001
Sex			
Males	75,418 (49.17)	258,296 (43.21)	< 0.001
Females	77,977 (50.83)	339,458 (56.79)	
Duration of consultation (Median, IQR)	9 (5–14)	7 (4–12)	< 0.001
Closure status			< 0.001
Completed successfully	74,917 (48.84)	271,039 (45.34)	
Cancelled	4,225 (02.75)	26,849 (04.49)	
Patient no-show	70,201 (45.76)	264,265 (44.21)	
Physician no-show	2,213 (01.44)	14,316 (2.39)	
Planned	404 (00.26)	3,649 (0.61)	
Not planned	1,244 (00.81)	16,536 (02.77)	
No action specified	192 (00.13)	1,106 (00.19)	

### Trend of Hospital-Based Virtual Consultations by Speciality vs. Home-Based Consultations Across Time

**Fig. 2:**
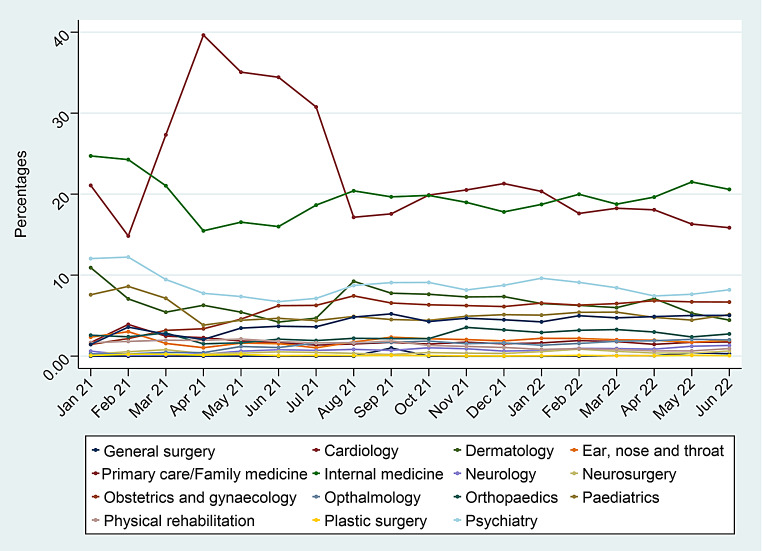
Time trend of virtual consultations between January 2021 and June 2022 in the Kingdom of Saudi Arabia

Figure 2 shows the trend of all specialities from the hospital-based consultations across the months from January 2021 to June 2022. Given that both primary care/family medicine and internal medicine were the most demanded specialities, the results show that both were also the most demanded across all 18 months of the study period. Demand for primary care/family medicine especially had risen during the months from March 2021 to July 2021 with a peak in April of 2021.

**Fig. 3:**
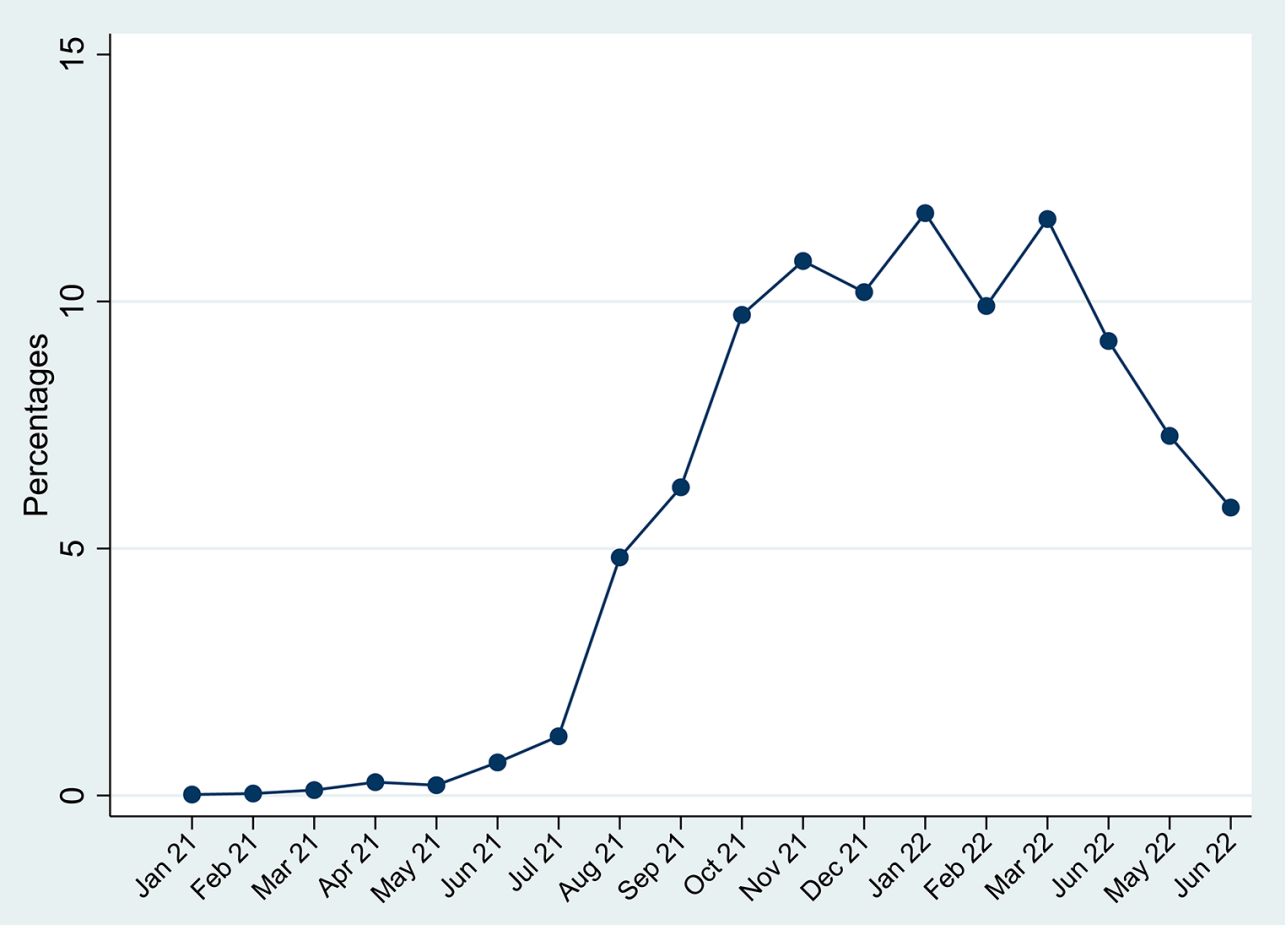
Time trend of home-based consultations between January 2021 and June 2022 in the Kingdom of Saudi Arabia

Figure 3 shows the trend of home-based consultations across the months from January 2021 to June 2022. The results show that a steady increase in the number of consultations is observed from August 2021 until May 2022, with two peaks in January and March of 2022.

### The Epidemiology of Home-Based Consultations

**Fig. 4:**
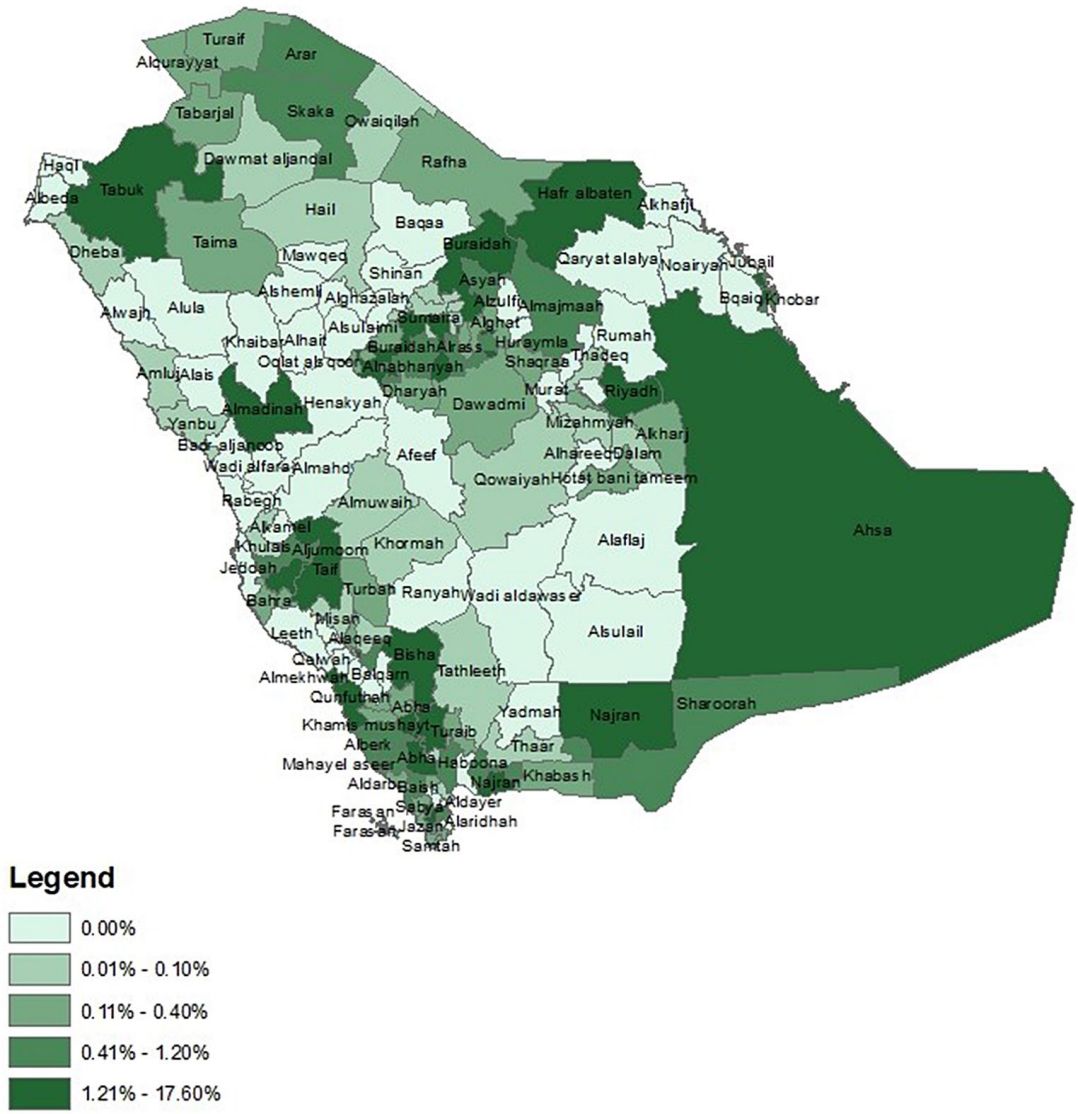
Distribution of home-based virtual consultations across the Kingdom of Saudi Arabia. Digitised map acquired from MapDev for Information Systems Technology. Administrative boundary data was obtained from the Saudi Ministry of Health

Figure 4 shows the epidemiology and distribution of home-based consultations across the governorates of the KSA. The main cities of each administrative area are observed to have a higher proportion of these consultations, such as the capital Riyadh, Madinah, Abha and Tabuk. However, other non-major cities are also seen to have a high proportion of these consultations such as Hafr Albaten, Bisha and Taif.

## Discussion

This study was successfully able to epidemiologically explore the pattern of VCs in its main two types in the KSA utilising secondary routinely collected data from the MoH spanning the entire country. It is also the first to provide empirical evidence of variability in use across all governorates of the country. The analyses and patterns currently presented have been able to shed light on the growing importance of primary care and family medicine in particular as well as identify areas of weakness warranting further exploration and assessment.

### Sociodemographic Patterns Across Both Types of Consultations

Most VCs were of the hospital-based type. These consultations are initiated by the treating physician which may be prompted either by a referral from the home-based VC, or from a physical primary care appointment, or as a follow-up from a previous physical or virtual appointment rendering this type to be more common than home-based consultations. Also, the fact that these are hospital-based would mean that a variety of specialities are involved ranging from family medicine to more complex specialities such as oncology and surgery requiring more appointments per patient. Similar to more telehealth informed places, family medicine is the most used specialty in hospital-based consultations for both initial visits and for follow ups as well [14, 15].

As observed elsewhere, primary care/family medicine and internal medicine were the leading specialities in VCs [15]. Surgical specialties and rehabilitation services were the least specialties in demand and in providing virtual care to patients. The need for physical attendance for the maximum benefit of these services might be a leading cause of such a finding.

Females tended to be more frequent users of virtual services than males. Results available from the very limited local studies were variable. One study that examined VCs across only six tertiary hospitals found similar findings of higher use among females but did not provide a justification to their findings [16]. Another study found that males were higher users, but it only included 528 participants and the sampling technique was a convenience sampling which indicates that the results are not generalisable [17]. Since the current study has used a full coverage of all consultations renders the results more reliable. The higher satisfaction of females towards the use of VCs as well as convenience due to transportation related issues may have contributed towards this finding [18].

The overall mean age of users of VCs in general was 38.50 years. This reflects that VCs users in the KSA are younger than that reported in the US and the UK [19]. Also, in a local comparative study, health applications’ users were generally younger than non-users. The KSA benefits from a much younger population. The latest census data of 2022 reported that the average age of the Saudi population is 29 years [20].

The median consultation time was found to be 8 min in general, but slightly longer for home-based consultations. These durations are generally longer than that reported elsewhere [21, 22]. Variations in duration between home-based and hospital-based consultations naturally varies based on several factors including patients’ age, sex, personal and family history and complaints. Specifically, for home-based consultations, physicians usually require more time due to the fact that in many cases physicians do not have access to patients’ medical records.

With family practice being the most utilised specialty virtually, it also has the greatest number of patient no-shows. This could be explained proportionally in the most part, but other factors may play a role as well. The higher age group that utilizes the service and the chronicity of the conditions that appointments are made for are major contributors to such observations [23].

### Distribution of Clinics Typically Part of a Primary Care Centre

A close examination of the services that are typically provided in primary care settings but have been provided during hospital-based consultations show that almost 80% of these services are actually family medicine clinics provided by consultants in family medicine within hospitals. This conclusion is essential for policymakers to track health service utilisation, evaluate workforce needs, and create a long-term strategy to meet those needs [24].

Furthermore, the vast majority of home-based consultations were actually either primary care or family medicine clinics. The general community is becoming more aware of their value and are more trusting of the services provided by them [25]. In line with this finding, we also found that almost half of all home-based consultations were successfully completed and closed, which is higher than successful closures for all other specialties combined. VCs could also address organizational concerns and accessibility problems for primary healthcare, which have been identified in the literature as the main obstacles to Saudi patients’ utilisation of primary care and family medicine facilities [26].

The current study found that services such as smoking cessation and health education were utilised more in hospital-based VCs. It may be that patients and their healthcare providers have agreed upon a follow-up plan for these consultations. Contrarily, maternity follow-ups were more common in home-based consultations. One of the main focuses of the Saudi health transformation strategy is women’s health, and epidemiological studies show that women health related problems are becoming more prevalent [27, 28]. Having qualified primary care staff who can handle women’s health issues by providing them with proper continuous medical education and training is crucial. The women’s health fellowship program for family physicians is well established in the KSA due to the increasing demands of this field [29]. These initiatives may therefore lessen the number of visits to the emergency department for non-urgent OB/GYN related issues [30, 31].

### The Epidemiology and Pattern of Virtual Consultations

The demand for family medicine hospital-based consultations increased at the start of the study, from March 2021 to July 2021. However, a steady rise in home-based consultations was seen which shows that the use of home-based consultations has in some regard compensated for the loss in hospital-based family medicine clinics. This may be attributed to the recently increased public awareness and acceptability of VCs [32].

The highest proportion of VCs were observed in the main governorates, including Riyadh, Ahsa, Hafer Albaten, Buraidah, Tabuk, Almadina, Taif, and Najran. Based on the 2017 national statistics, Riyadh, Almadina, Jazan, Qaseem, and the Eastern governorates were the top five regions with the number of manpower in primary healthcare centres [33]. This might explain the availability of physicians to provide and manage online services in these regions. In Riyadh in 2019, the majority of physicians believed in VCs and were willing to adopt them [34]. This suggests that the readiness of healthcare providers may be one of the predominant reasons for the successful adoption of VCs in the KSA. Furthermore, these particular areas are highest in socioeconomic levels compared to rural areas based on the AlOmar socioeconomic indices [35].

The current study also shows that the lowest proportions were in rural areas such as Alula, Alaflaj, Ranyah and Alsulail. Along with differences in socioeconomic levels between urban and rural areas, the lower use of VCs in these rural areas may be due to lower levels of awareness of VCs, as well as limited access to the internet and technology infrastructure. Additionally, cultural factors and preference for face-to-face consultations may also play a role, which has been particularly highlighted in the western governorates, where non-users in those areas had previously reported their preference for hospital emergency departments rather than VCs [17].

### Implications for Health Policy and Recommendations for Future Research

Despite the surge in virtual care adoption [36], our utilisation numbers of the service in general remains on the lower side of the international average among the primary care/family medicine specialty [37]. In countries like Canada and Australia, long standing telehealth services makes its use more relevant to the population, whereas, in our region, the peak of service utilisation was during the crisis of the COVID-19 pandemic [38]. The adoption of VCs in the KSA has shown promising results, particularly in urban areas where access to healthcare providers and technology is more readily available. Efforts should be made to promote the use of VCs in rural areas, as it can improve access to healthcare services and reduce the burden on healthcare facilities. Continued efforts are needed to educate and raise awareness among the population about the benefits and convenience of VCs.

The extremely large proportion of patient no-shows warrants further investigation. Patient no-shows are missed timeslots which have been shown to negatively impact the utilisation of healthcare resources. Several causes of patient no-shows are immeasurable, however, through unified electronic patient records, patterns of no-shows maybe detected which in turn may help to predict patients who are more likely to miss appointments [39].

### Limitations

Although we were able to analyse over a million consultations, we were only able to analyse the variables collected by the MoH. Therefore, specific detailed variables such as the patients’ conditions, comorbidities, number of referrals and follow-ups were not analysed due to their unavailability. Furthermore, reasons for no-shows were not available, so we were not able to explore this issue further.

## Conclusion

This study epidemiologically described the pattern of both hospital-based and home-based VCs in the KSA. Family medicine consultations were predominant even in hospital-based settings which further highlight the importance of this speciality. Maternity services were highly adopted in home-based consultations. Also, adoption of VCs was found to be higher in governorates with higher socioeconomic levels. The high proportion of no-shows warrants further investigation.

## Data Availability

The data that support the findings of this study are available from the Ministry of Health, but restrictions apply to the availability of these data, which were used under license for the current study, and so are not publicly available. Data are however available from the authors upon reasonable request and with permission of the Ministry of Health.
